# Case Report: Intramedullary solitary fibrous tumor at the C7-T1 level diagnosed by STAT6 and treated with maximal safe resection

**DOI:** 10.3389/fsurg.2026.1845706

**Published:** 2026-06-02

**Authors:** Changli Han, Zixiao Li, Zhang Xiong, Mengwei Ma, Weicheng Wang, Yuqing Wang, Guangfu Di, Xiaochun Jiang

**Affiliations:** 1 Department of Neurosurgery, The First Affiliated Hospital of Wannan Medical College, Yijishan Hospital of Wannan Medical College, Wuhu, China; 2 Anhui Digital Brain Engineering Research Center, The First Affiliated Hospital of Wannan Medical College, Yijishan Hospital of Wannan Medical College, Wuhu, China

**Keywords:** case report, intramedullary, neurosurgery, solitary fibrous tumor, spinal cord, STAT6

## Abstract

Intramedullary solitary fibrous tumor is an exceptionally rare spindle-cell neoplasm of the central nervous system and is rarely diagnosed before surgery because the clinical presentation and imaging findings are non-specific. We report a man in his early 60s with more than 7 years of progressive bilateral lower-extremity numbness that worsened during the month before admission without an identifiable precipitating event. Magnetic resonance imaging demonstrated an enhancing intramedullary lesion at the C7-T1 level. The patient underwent microscopic tumor resection through a posterior C7-T2 laminectomy under intraoperative ultrasound guidance and multimodal intraoperative neurophysiological monitoring, including somatosensory evoked potentials, transcranial motor evoked potentials, D-wave monitoring, and free-running electromyography. Gross total resection was achieved without new neurological deficit. Histology showed spindle cells in fascicular and storiform arrangements with branching staghorn-like vessels. Immunohistochemistry demonstrated nuclear STAT6 positivity with co-expression of CD34, CD99, and Bcl-2. Mitotic activity was low, with only 2–3 mitoses per 10 high-power fields, and no definite tumor necrosis was identified, supporting the diagnosis of a CNS WHO grade 1 solitary fibrous tumor. Sensory symptoms improved after surgery, and no recurrence was detected on magnetic resonance imaging at 18-month follow-up. This short-term outcome is encouraging, but long-term radiological surveillance remains necessary.

## Introduction

Solitary fibrous tumors are uncommon mesenchymal neoplasms that were first recognized in the pleura but are now known to occur in many anatomical locations. Primary central nervous system solitary fibrous tumors are rare, and spinal lesions represent only a minority of these tumors. Most spinal lesions are dural-based or intradural extramedullary, whereas tumors arising within the spinal cord parenchyma are distinctly unusual (1–3). True intramedullary spinal solitary fibrous tumor is even rarer. Because older reports variably used the terms solitary fibrous tumor and hemangiopericytoma and many cases predated routine STAT6 immunohistochemistry, the exact number of unequivocal true intramedullary cases remains difficult to define. The available literature mainly consists of isolated case reports and very small reports, highlighting the diagnostic and surgical value of each well-documented case (2, 4).

Intramedullary solitary fibrous tumor remains challenging for neurosurgeons because the symptoms are usually caused by slow spinal cord compression and lack pathognomonic features. Likewise, magnetic resonance imaging often shows an enhancing lesion that overlaps with more common diagnoses such as ependymoma, schwannoma, or meningioma (5). Definitive diagnosis therefore depends on histopathology and immunohistochemistry, particularly nuclear STAT6 expression, which is a practical surrogate for the NAB2-STAT6 fusion that characterizes this tumor (6–8). We present a case of intramedullary solitary fibrous tumor at the C7-T1 level and focus on the features most relevant to surgical planning, pathological confirmation, and postoperative surveillance.

## Case description

A man in his early 60s presented with bilateral lower-extremity numbness that had been present for more than 7 years and had worsened during the month before admission. No definite precipitating event, including trauma, infection, fever, acute systemic illness, or recent spinal procedure, was identified. During the months preceding hospitalization, the sensory disturbance progressed upward to involve the chest and back. He had no history of prior spinal surgery, malignancy, or major trauma. Neurological examination showed preserved motor strength in all extremities, mild bilateral lower-extremity numbness, diminished reflexes, and no definite pathological signs. He was ambulatory without objective gait disturbance, and no sphincter dysfunction, severe axial pain, or radicular pain was documented. The preoperative modified McCormick grade was I.

Preoperative cervical magnetic resonance imaging demonstrated a focal enhancing intramedullary lesion at the C7-T1 level, measuring approximately 20 × 12 × 10 mm. The lesion was slightly hypointense on T1-weighted sequences and heterogeneously hyperintense on T2-weighted sequences, with marked heterogeneous enhancement after gadolinium administration. On axial contrast-enhanced images, the lesion was centered within the spinal cord parenchyma and caused focal cord expansion and deformation. Coronal and sagittal images confirmed that the mass was centered at the cervicothoracic junction and remained confined to the intramedullary compartment. No definite extramedullary component or dural attachment was identified on the available images. There was no obvious syrinx, cystic change, intratumoral hemorrhage, or prominent flow voids. Although these imaging findings supported an intramedullary neoplasm, they were not specific enough to distinguish solitary fibrous tumor from ependymoma or other more common intramedullary tumors (Figure 1). The clinical course and key diagnostic and therapeutic milestones are summarized in Table 1.

**Figure 1:**
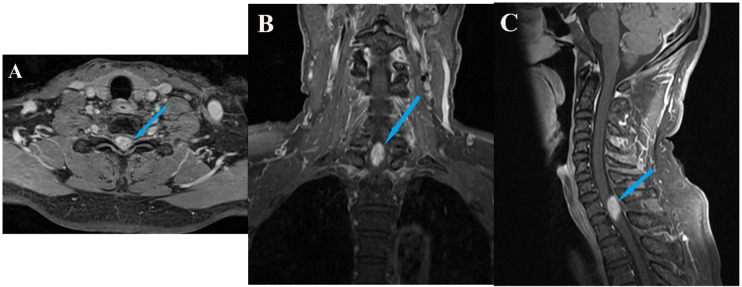
Preoperative cervical magnetic resonance imaging. Axial **(A)**, coronal **(B)**, and sagittal **(C)** contrast-enhanced T1-weighted images show a focal enhancing intramedullary lesion at the C7-T1 level. The lesion is centered within the spinal cord parenchyma, causes focal cord expansion and deformation, and shows marked heterogeneous enhancement. No definite extramedullary component or dural attachment is evident on these images.

**Table 1:** Timeline of the episode of care.

Time point	Clinical course
>7 years before admission	Onset of bilateral lower-extremity numbness.
1 month before admission	Progressive worsening of numbness with cephalad extension to the chest and back.
Admission	Neurological examination showed preserved strength, bilateral lower-extremity numbness, diminished reflexes, no sphincter dysfunction, and modified McCormick grade I.
Preoperative imaging	Contrast-enhancing intramedullary C7-T1 lesion measuring approximately 20 × 12 × 10 mm, with focal cord expansion and no definite extramedullary component or dural attachment.
Index surgery	Microscopic posterior C7-T2 laminectomy with intraoperative ultrasound and multimodal intraoperative neurophysiological monitoring, including somatosensory evoked potentials, transcranial motor evoked potentials, D-wave monitoring, and free-running electromyography; gross total resection based on intraoperative microscopic assessment.
Postoperative day 7	Ambulation resumed without new motor deficit.
Postoperative day 17	Discharged without adjuvant radiotherapy or chemotherapy.
18-month follow-up	Further sensory improvement, modified McCormick grade I, and no radiological recurrence on magnetic resonance imaging.

## Diagnostic assessment

The preoperative differential diagnosis included ependymoma, schwannoma, meningioma, and other spindle-cell or vascular lesions. No single imaging sign confidently favored solitary fibrous tumor. Given the intramedullary location and the non-specific imaging appearance, the preoperative strategy required careful weighing of a diagnostic biopsy vs. upfront maximal safe resection. Intraoperative frozen section suggested a mesenchymal neoplasm, but final diagnosis relied on permanent sections and immunohistochemistry.

Microscopically, the lesion was composed of spindle cells arranged in fascicular and storiform patterns with branching staghorn-like vessels. Mitotic activity was low, with only 2–3 mitoses per 10 high-power fields in the most mitotically active areas. No definite tumor necrosis was identified. Immunohistochemistry showed positivity for STAT6, CD34, CD99, and Bcl-2, with a Ki-67 labeling index of approximately 5%; AE1/AE3, EMA, and S-100 were negative (Figure 2). Taken together, these findings supported the diagnosis of CNS WHO grade 1 solitary fibrous tumor.

**Figure 2:**
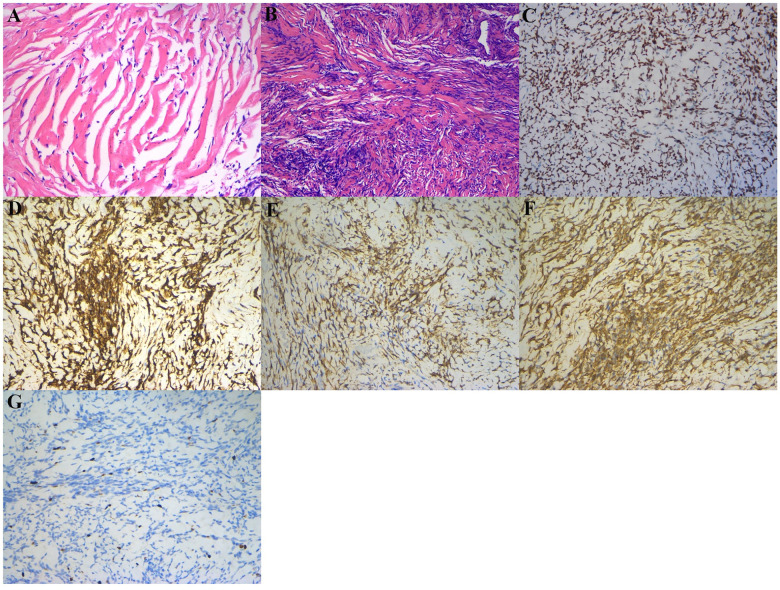
Histopathological and immunohistochemical findings. **(A,B)** Hematoxylin and eosin staining shows spindle-cell proliferation with a storiform arrangement and staghorn-like vessels. **(C–F)** Immunohistochemistry shows positive staining for STAT6, CD34, Bcl-2, and CD99. Ki-67 shows a proliferation index of approximately 5% **(G)**.

## Therapeutic intervention, follow-up and outcomes

The patient underwent microscopic resection of the C7-T1 intramedullary tumor through a posterior C7-T2 laminectomy under general anesthesia. After removal of the spinous processes, the spinal canal was opened using a high-speed drill to expose the dura over the C7-T2 region. Before durotomy, intraoperative ultrasound was used to localize the intramedullary mass and confirm its cranio-caudal extent, thereby guiding the level of dural opening and myelotomy. Given the long symptom duration, recent progression, and the focal surgically accessible lesion, maximal safe resection was considered more appropriate than simple biopsy alone. After durotomy, the lesion was accessed through a posterior median sulcus myelotomy. The lesion was identified within the spinal cord parenchyma and appeared gray-white, moderately vascular, and relatively firm. Although the tumor was not encapsulated and was poorly demarcated in some areas, a partially identifiable tumor-cord interface could be developed based on differences in color, consistency, and vascularity.

Multimodal intraoperative neurophysiological monitoring was used throughout the procedure, including somatosensory evoked potentials, transcranial motor evoked potentials, D-wave monitoring, and continuous free-running electromyography. D-wave monitoring was used to assess corticospinal tract integrity during intramedullary tumor manipulation, while free-running electromyography was recorded from C8-T1-innervated upper-limb muscle channels because the C7-T2 posterior exposure and cervicothoracic tumor location carried a potential risk of C8-related iatrogenic injury. During tumor manipulation and resection, no sustained reduction in somatosensory or motor evoked potentials, no clinically significant D-wave amplitude loss, and no persistent neurotonic free-running electromyography discharges were observed. Circumferential dissection and piecemeal internal debulking allowed removal of all macroscopically visible tumor while preserving spinal cord function (Figure 3). Because early postoperative MRI was not available, gross total resection was defined according to intraoperative microscopic assessment, with no visible residual tumor at the end of surgery, and was further supported by follow-up MRI.

**Figure 3:**
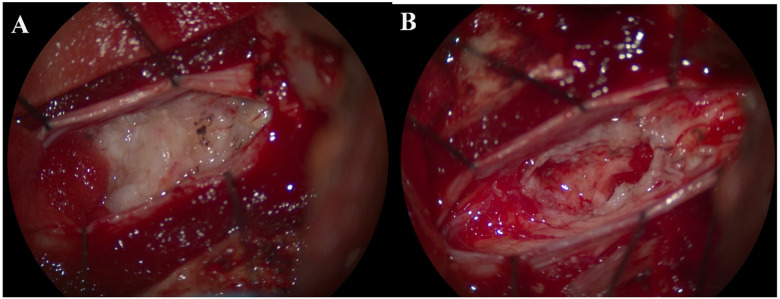
Intraoperative findings. **(A)** The tumor arose from within the spinal cord and showed an indistinct interface with the surrounding parenchyma. **(B)** Operative field after tumor removal.

Postoperatively, the patient developed no new motor deficit, gait deterioration, or sphincter dysfunction and reported improvement in bilateral lower-extremity numbness. He began ambulation on postoperative day 7 and was discharged on postoperative day 17. The modified McCormick grade remained I at discharge. No adjuvant radiotherapy or chemotherapy was administered. At 18-month follow-up, the patient remained independently ambulatory, sensory symptoms had further improved, and follow-up magnetic resonance imaging from an outside institution showed no evidence of residual or recurrent disease. The modified McCormick grade remained I at the last follow-up.

## Discussion

This case highlights a clinically important but uncommon diagnostic problem in spinal surgery. Intramedullary solitary fibrous tumor is rare, and the preoperative diagnosis is difficult because both the symptoms and imaging features overlap with more common intramedullary lesions (2, 3, 5). At the cervicothoracic junction, even a small lesion may produce long-standing but initially subtle myelopathic symptoms, which can delay diagnosis.

From a pathological standpoint, the current case illustrates the practical value of combining morphology with immunohistochemistry. The spindle-cell architecture and branching vascular pattern suggested solitary fibrous tumor, but these features alone are not fully specific. Nuclear STAT6 expression remains the most useful confirmatory marker in routine practice because it reflects the defining NAB2-STAT6 fusion. Beyond its diagnostic value, STAT6 also has broader biological significance because it links routine immunohistochemistry to the molecular pathogenesis of solitary fibrous tumor and helps distinguish this entity from histological mimics with different clinical behavior. Although STAT6 positivity by itself is not a validated adverse prognostic marker, its identification is clinically meaningful because it places the lesion within the modern molecular framework of solitary fibrous tumor, which is relevant when considering recurrence risk, long-term surveillance, and interpretation of the limited spinal literature (7, 9). According to the 2021 World Health Organization classification of central nervous system tumors, CNS SFT grading is mainly determined by mitotic activity and tumor necrosis. In the present case, the mitotic count was only 2–3 per 10 high-power fields and no definite tumor necrosis was identified, supporting the diagnosis of CNS WHO grade 1 SFT (6). CD34, CD99, and Bcl-2 are supportive but less specific markers and should be interpreted in the context of the full histological picture (8, 10).

The operative findings were also instructive. In this patient the tumor arose within the spinal cord parenchyma and had an indistinct interface with the surrounding cord in some areas, which increases the risk of neurological injury during resection. For this reason, maximal safe resection rather than aggressive resection at any cost should be the operative goal. At the same time, the choice between limited biopsy and definitive resection should be individualized through multidisciplinary decision making. For deeply seated or poorly accessible intramedullary lesions with a high anticipated neurological risk, biopsy may be a reasonable strategy to establish the diagnosis before further treatment planning. However, when the lesion is focal, surgically reachable, and associated with progressive symptoms, maximal safe resection may provide both tissue diagnosis and therapeutic decompression in a single procedure.

In our case, intraoperative ultrasound was helpful before durotomy to localize the lesion and define the operative corridor, while multimodal intraoperative neurophysiological monitoring supported function-preserving microsurgery. D-wave monitoring provided continuous information regarding corticospinal tract integrity, and free-running electromyography was relevant because of the potential risk of C8-related injury during exposure and manipulation at the cervicothoracic junction. More broadly, intraoperative ultrasound may also assist assessment of residual lesion or adequacy of decompression before closure (11–13). The wording of gross total resection should be interpreted as removal of all macroscopically visible tumor under the operative microscope, supported by the absence of residual or recurrent tumor on follow-up MRI, rather than by immediate postoperative MRI. This experience is consistent with larger spinal series showing that extent of resection remains central to disease control whenever neurologically feasible (1, 3, 14).

The current report also supports long-term surveillance even when the lesion is histologically low grade. The absence of radiological recurrence at 18 months is encouraging, but this interval is too short to establish durable disease control in solitary fibrous tumor. In a multicenter series and systematic review, recurrence was observed in 43% of spinal SFT cases after a mean interval of 5.8 years, with reported recurrence intervals extending up to 25 years (1). A recent report further described recurrence of spinal cord SFT 19 years after gross total resection and emphasized the need for lifelong follow-up (4). Published central nervous system series have also reported uncertainty regarding the benefit of adjuvant radiotherapy after complete resection, especially for low-grade disease, so routine postoperative radiotherapy cannot be recommended on the basis of currently available evidence (14, 15). Accordingly, our management strategy after gross total resection was clinical follow-up plus periodic magnetic resonance imaging.

For residual, recurrent, metastatic, or progressive disease, radiotherapy or radiosurgery may be considered on a case-by-case basis within a multidisciplinary setting (16, 17). Although evidence specifically for intramedullary spinal SFT remains limited, Wang et al. recently analyzed 17 cases of extracranial metastases from intracranial SFTs and emphasized that adjuvant radiotherapy may delay recurrence and that Gamma Knife radiosurgery can be effective for small recurrent lesions (17). In the spinal cord, however, radiation-based treatment must be balanced against the risk of injury to surrounding functional neural tissue. In this confined anatomical environment, emerging radiobiological adjuncts, including radioenhancers, may represent a promising strategy to improve the therapeutic ratio by enhancing tumor response while helping to preserve adjacent healthy tissue (18). Their application to spinal solitary fibrous tumor remains investigational and should not be interpreted as a routine indication in the present case. Nevertheless, if residual or recurrent disease is detected during long-term surveillance, salvage radiotherapy or stereotactic radiosurgery should be discussed within a multidisciplinary framework.

The main strengths of this report are the clear intramedullary origin, the detailed operative description, the complete immunohistochemical confirmation, and the follow-up showing short-term radiological control. The main limitation is the relatively short follow-up interval, which does not permit conclusions about long-term tumor behavior. The principal lesson from this report is that, although rare, solitary fibrous tumor should remain in the differential diagnosis of enhancing intramedullary lesions and that definitive diagnosis depends on careful pathological workup, especially STAT6 immunostaining.

## Patient perspective

The patient reported that surgery relieved the long-standing numbness in both legs and that no new weakness occurred after the operation. He was satisfied with the treatment and agreed with continued long-term magnetic resonance imaging follow-up because of the possibility of delayed recurrence.

## Data Availability

The original contributions presented in the study are included in the article/Supplementary Material, further inquiries can be directed to the corresponding author.
